# High levels of the MDM2 oncogene in paediatric rhabdomyosarcoma cell lines may confer multidrug resistance

**DOI:** 10.1054/bjoc.2001.2145

**Published:** 2001-11

**Authors:** H A Cocker, S M Hobbs, N Tiffin, K Pritchard-Jones, C R Pinkerton, L R Kelland

**Affiliations:** CRC Centre for Cancer Therapeutics, The Institute of Cancer Research, Cotswold Road, Sutton, Surrey, SM2 5NG, UK; Section of Paediatrics, The Institute of Cancer Research/Royal Marsden NHS Trust, Cotswold Road, Sutton, Surrey, SM2 5NG, UK

**Keywords:** *mdr-1*, mdm2, resistance, P-glycoprotein, rhabdomyosarcoma

## Abstract

The MDM2 protein is known to be overexpressed in some sarcomas including rhabdomyosarcoma. However, the extent to which the MDM2 protein influences sensitivity to chemotherapeutic drugs is unclear. We have analysed this further using stable transfection of the *mdm2* gene into 4 well-characterised human paediatric rhabdomyosarcoma cell lines. Transfection with the *mdm2* gene resulted in increased levels of the MDM2 protein in all the cell lines. In 2 of the lines, SCMC and RD, the *mdm2* gene caused between 2-fold and 61-fold increase in resistance to vincristine, etoposide and doxorubicin but not to cisplatin. In these lines there was an increase in expression of the *mdr-1* gene which encodes P-glycoprotein, but not the *mrp1* gene which encodes the multidrug resistance protein (MRP). The resistance was reversible using the MDR modulator PSC833, confirming the presence of P-glycoprotein. We conclude that MDM2 overexpression may be a mechanism by which multidrug resistance is regulated in some rhabdomyosarcomas. © 2001 Cancer Research Campaign http://www.bjcancer.com


*British Journal of Cancer* (2001) **85**(11), 1746–1752
© 2001 Cancer Research Campaign
doi: 10.1054/ bjoc.2001.2145, available online at http://www.idealibrary.com on IDEAL

### High levels of the MDM2 oncogene in paediatric rhabdomyosarcoma cell lines may confer multidrug resistance

**HA Cocker[1], SM Hobbs[1], N Tiffin[2], K Pritchard-Jones[2], CR Pinkerton[2] and LR Kelland[1]**

[1]CRC Centre for Cancer Therapeutics, The Institute of Cancer Research, Cotswold Road, Sutton, Surrey, SM2 5NG, UK; [2]Section of Paediatrics, The Institute of Cancer Research/Royal Marsden NHS Trust, Cotswold Road, Sutton, Surrey, SM2 5NG, UK

**Summary** The MDM2 protein is known to be overexpressed in some sarcomas including rhabdomyosarcoma. However, the extent to which the MDM2 protein influences sensitivity to chemotherapeutic drugs is unclear. We have analysed this further using stable transfection of the *mdm2* gene into 4 well-characterised human paediatric rhabdomyosarcoma cell lines. Transfection with the *mdm2* gene resulted in increased levels of the MDM2 protein in all the cell lines. In 2 of the lines, SCMC and RD, the *mdm2* gene caused between 2-fold and 61-fold increase in resistance to vincristine, etoposide and doxorubicin but not to cisplatin. In these lines there was an increase in expression of the *mdr-1* gene which encodes P-glycoprotein, but not the *mrp1* gene which encodes the multidrug resistance protein (MRP). The resistance was reversible using the MDR modulator PSC833, confirming the presence of P-glycoprotein. We conclude that MDM2 overexpression may be a mechanism by which multidrug resistance is regulated in some rhabdomyosarcomas. © 2001 Cancer Research Campaign http://www.bjcancer.com

**Keywords**: *mdr-1*; mdm2; resistance; P-glycoprotein; rhabdomyosarcoma

Rhabdomyosarcoma is a malignant soft-tissue sarcoma that accounts for 6–7% of all cancers in children (Enzinger and Weiss, 1995). Drugs commonly used to treat this disease include the vinca alkaloids, anthracyclines, etoposide and cyclophosphamide. Initial treatment is often successful but relapse due to drug resistance remains a major obstacle to survival. In some, but not all, studies the expression of P-glycoprotein (P-gp, encoded by the *mdr-1* gene) in clinical samples has been associated with adverse prognosis (Chan et al, 1990; Kuttesch et al, 1996). Our more recent in vitro studies have shown that relatively low levels of P-gp and the multidrug resistance-related protein (MRP) may contribute to resistance (Cocker et al, 2000).

The MDM2 protein is often overexpressed in sarcomas and has been shown to be overexpressed in some rhabdomyosarcoma cell lines in vitro (Keleti et al, 1996; Cocker et al, 2000; Taylor et al, 2000) and occasionally (approximately 10% of cases) in patient tumour specimens (Meddeb et al, 1996; Taylor et al, 2000). The MDM2 protein binds and inactivates the tumour suppressor protein P53 and in turn p53 is a transcriptional trans-activator of MDM2. Mutant p53 has also recently been shown to stabilise MDM2 (Peng et al, 2001). It also interacts with proteins such as p19-ARF and Rb to control cellular growth (Ashcroft and Vousden, 1999). As well as a link being shown between p53 and *mdr-1* (Chin et al, 1992; Thottassery et al, 1997), a direct link may exist between the expression of MDM2 and the expression of *mdr-1*. In one example, transfection of a glioblastoma line with the *mdm2* gene resulted in increased expression of both the *mdr-1* gene and functionally active P-gp (Kondo et al, 1996). However, the absence of data for appropriate empty vector controls in this study does not exclude the possibility that the increased expression of *mdr-1* resulted from the transfection process itself. In addition, it is unclear whether the increase was mediated by interaction of MDM2 with P53, which may affect expression of *mdr-1* (Li et al, 1997). Alternatively, there may be direct activation of transcription by MDM2 (Leveillard and Wasylyk, 1997). There are also clinical studies which have linked expression of MDM2 with resistance to doxorubicin in acute lymphoblastic leukaemia (Zhou et al, 1997) and breast carcinoma (Suzuki et al, 1998) but conversely, expression of *mdm2* mRNA has been correlated with increased duration of survival in ovarian cancer (Tanner et al, 1997).

This study employed stable transfection of *mdm2* into human rhabdomyosarcoma (RMS) cell lines to address whether (a) MDM2 regulates the expression of MDR genes in rhabdomyosarcoma and (b) whether this confers multidrug resistance. We have used 4 well characterised human rhabdomyosarcoma cell lines with lines selected on the basis of previous experiments determining constitutive expression of P53, MDM2 and P-gp (Cocker et al, 2000). Of the 4 lines only one, SCMC, has wild-type P53 function. The remaining lines, Rh30, RMS and RD all have point mutations in the *p53* gene although some evidence of P53 function (up-regulation of P21 following irradiation) has been demonstrated in RMS (Cocker et al, 2000).

### MATERIALS AND METHODS

### Cell lines

This study uses 4 rhabdomyosarcoma cell lines, as described previously (Cocker et al, 2000). Rh30 (mutant p53) was a gift

*Received 25 May 2001*
*Revised 22 August 2001*
*Accepted 10 September 2001*

*Correspondence to:* LR Kelland from Dr PJ Houghton (St Judes Children's Research Hospital). SCMC (p53 wild-type) was a gift from T Sawada (Kyoto Prefectual University of Medicine, Japan). RMS and RD (both mutant p53) were obtained from the American Type Culture Collection (Manassas, VA, USA). The human ovarian carcinoma cell lines CH1 and its multidrug resistant variant CH1DoxR were used as negative and positive controls for *mdr-1* and P-gp expression (Sharp et al, 1994). The human non-small-cell lung carcinoma cell line CORL23 and its multidrug resistant variant CORL23/R were used as negative and positive controls for *mrp1* expression (Barrand et al, 1994).

All cell lines were maintained in RPMI 1640 medium (Sigma, Poole, Dorset, UK) supplemented with 10% fetal bovine serum (Life Technologies, Scotland, UK) and 2 mM L-glutamine (Sigma). Cells were grown as attached monolayers and were incubated at 37˚C in a humidified atmosphere with 5% $CO_2$. All cell lines were routinely screened for *Mycoplasma* by PCR assay (Stratagene, Cambridge, UK).

### Drugs and chemicals

Vincristine (David Bull Laboratories) and Etoposide (Bristol Myers Squibb Pharmaceuticals) stock solutions were obtained as pharmacy preparations at concentrations of 1.083 mM and 34 mM respectively. Doxorubicin (Sigma) was dissolved in water to give a stock solution of 1 mM. Cisplatin (Johnson Matthey Technology Centre, Reading, UK) was dissolved in 0.9% saline to give a stock solution of 1 mM. PSC833 (Novartis, Basel, Switzerland) was dissolved in absolute ethanol to give a 1 mM stock. All these drugs were stored at –20˚C with the exception of vincristine which was stored at 4˚C. Immediately before use the drugs were diluted with RPMI 1640 medium prepared as for cell culture, with the exception of vincristine which was diluted in RPMI medium without any supplements.

MTT (3-(4,5-dimethylthiazol-2yl)-2,5-diphenylterazolium bromide) (Sigma) was dissolved in PBS to produce a stock solution of 5 mg $ml^{-1}$ which was stored at 4˚C. Puromycin (Sigma) was dissolved in water to give a stock solution of 250 µg $ml^{-1}$ and stored at –20˚C.

### Transfection

The plasmid vector pEFIRES-P (also known as F373) contains the puromycin resistance gene (*pac*) and the gene of interest within a single transcription cassette designed to produce a bicistronic message (Hobbs et al, 1998). A cDNA containing the human *mdm2* coding sequence (originally obtained from Dr B Vogelstein) was cloned into the multiple cloning site of F373, resulting in the vector F407. Cell lines were transfected with F373 or F407 by DOTAP lipofection (Roche Diagnostics Ltd, Lewes, E Sussex, UK) using a 6 hour incubation with 5 µg of vector DNA complexed with 30 µl DOTAP. The cells were then incubated in growth medium for 48 hours to recover and then exposed to puromycin at 0.3–0.5 µg $ml^{-1}$, a concentration pre-determined to kill all non-transfected cells. Surviving colonies were selected after 3 weeks and analysed for MDM2 expression by immunoblotting. Puromycin selection was maintained until one week before experiments.

### Immunoblotting

Immunoblotting was carried out as described previously (Cocker et al, 2000). The antibody IF-2 (Oncogene, Cambridge, MA, USA) was used to detect MDM2, C219 (Centocor, USA) was used to detect PgP from membrane preparations, DO-1 (Santa Cruz) was used to detect p53 and B-1-5-2 (Sigma) was used to detect α-tubulin to ensure equal loading of wells in all experiments.

### Growth inhibition assay

The MTT (3-(4,5-dimethylthiazol-2-yl)-2,5-diphenyltetrazolium bromide) assay (Mosmann, 1983), using 4 days continuous exposure, was used to measure growth inhibition, as previously described (Cocker et al, 2000). The effects of the modulator PSC833 on growth inhibition were also tested by this method. PSC833 was added immediately before addition of the cytotoxic agent at a concentration which kills less than 10% of cells (2 µM).

### Clonogenic assay

A clonogenic assay was used to measure cell survival. Briefly, cells were added to 6-well plates at concentrations of between $1 \times 10^3$ and $5 \times 10^3$ cells per well. The cytotoxic agents doxorubicin, vincristine, etoposide and cisplatin were added 24 h later. The cells were then incubated for a further 5 days after which the drug was removed. The experiment was terminated after a further 4–8 days, dependent on the cell line used. Surviving colonies were stained using methylene blue (0.5% methylene blue in 50% methanol) (Sigma) for 15 minutes, air dried and counted. The number of surviving colonies of greater than approximately 50 cells was expressed as a percentage of the number of colonies surviving in the absence of drug. Plating efficiency was between 4–10%.

### Real-time PCR

Relative expression of *mdr-1* and *mrp1* genes was measured using Taqman Real-time PCR (PE Applied Biosystems, Foster City, CA, USA), using the ABI Prism 7700 sequence detection system. 1 µg of total RNA was reverse transcribed in a 20 µl volume using Superscript reverse transcriptase (Life Technologies, Paisley, UK) and random hexanucleotides (Amersham Pharmacia Biotech UK Ltd, Bucks, UK), according to the manufacturer's instructions. Real-time PCR for *mdr-1* was performed in a final volume of 25 µl containing 1X TaqMan® Universal PCR Master Mix (PE Biosystems), 900 nM forward primer (5′-AAAAGTGAAAA-AGATAAGAAGGAAAAGAAA-3′), 900 nM reverse primer (5′-CACCATATACAACTTGTCAAGCCAA-3′) and 150 nM FAM/TAMRA dual-labelled oligonucleotide probe (5′-FAM-TTGAATAGCGAAACATTGAAAATACACTGACAGTTG-TAMRA-3′). Real-time PCR for *mrp1* was performed in a final volume of 25 µl containing 1X TaqMan® Universal PCR Master Mix, 300 nM forward primer (5′-AGATGACACCTCTCAA-CAAAACCA-3′), 300 nM reverse primer (5′-AGGTCTGC-CCAGCAGACG-3′) and 50 nM FAM/TAMRA dual-labelled oligonucleotide probe (5′-FAM- AACTGCCTTGGGATTTTTG-CTGTGGA-TAMRA-3′). Both primer and probe sets amplified across an exon/exon boundary, to prevent amplification of

*© 2001 Cancer Research Campaign* 

genomic DNA. 0.1 µl of cDNA was used in each reaction. To measure the total amount of sample RNA present, levels of 18S rRNA were quantitated in each reaction using TaqMan® Ribosomal RNA Control Reagents with a VIC/TAMRA oligonucleotide probe (PE Biosystems). Relative expression of *mdr-1* and *mrp1* was calculated from standard curves generated from cell lines CH1DoxR and CORL23/R respectively.

### Cloning of mrp1 cDNA

PCR amplification of cDNA from Rh30 cells was used to produce a 140 bp fragment of the *mrp1* gene. The primers were as previously published (Bordow et al, 1994). The amplified fragment was ligated into the pCR-Script™ Cam cloning vector (Stratagene) according to the manufacturer's instructions. The clones obtained were sequenced to check insert size, sequence and orientation. The plasmid was then digested using PvuII to generate a 588 bp fragment to use as a template.

### RNAse protection

α-$^{32}$P-labelled RNA transcripts complementary to actin, *mdr-1* and *mrp1* cDNA sequences were transcribed using a T7 polymerase MAXIscript kit (Ambion, Austin, TX, USA). The templates used were pTRI-β-actin-125-Human (Ambion), pTRI-*MDR-1* (Ambion) and the MRP template generated as described above. The resulting probes were purified on a denaturing 5% polyacrylamide gel. Hybridisation of probes and sample RNA was carried out using the RPA III kit (Ambion). The resulting protected fragments and unprotected controls were separated by electrophoresis using the QuickPoint nucleic acid separation system (Novex, San Diego, CA, USA). Bands were visualised using a phosphorimager (Molecular Dynamics, Sevenoaks, UK).

### Statistical analysis

Where appropriate, statistical significance was tested using a 2-tailed Student's *t*-test.

### RESULTS

The cell lines were transfected with the F407 construct which includes the full-length human *mdm2* gene. This resulted in increased levels of the 90 kDa MDM2 protein in all 4 cell lines when compared to the parental (w.t.) line or the F373 empty vector control (Figure 1). Various clones were selected and one (of very similar phase contrast morphology and doubling time) chosen for initial sensitivity determinations. Transfection with the empty vector control F373 occasionally also appeared to result in a small increase in MDM2 expression in 2 cell lines, SCMC and RD (as shown in Figure 1), whereas there was no change in Rh30 and RMS. The doubling times of the selected transfected cell lines were not significantly different from the parent cell lines (data not shown).

A growth inhibition assay was used to measure the sensitivity of the cell lines (parent line and a representative F373 empty vector and F407 *mdm2* clone of each) to the commonly used agents cisplatin, doxorubicin, etoposide and vincristine (Figure 2A–D, respectively). Transfection of the cell line RD (*p53* mutant) with the F407 vector resulted in significantly decreased sensitivity to all 4 agents. The decrease was particularly marked with vincristine (61.1-fold), doxorubicin (6.4-fold) and etoposide (4.8-fold). The F407-transfected SCMC line (*p53* wild type) also showed significantly decreased sensitivity to vincristine (5.8-fold), doxorubicin (5.4-fold) and etoposide (2.4-fold), but not to cisplatin. In contrast, no significant changes in sensitivity were seen with any agent in RMS, which constitutively overexpresses MDM2. In Rh30 there was a significant decrease in sensitivity to doxorubicin, but this was lower (1.5-fold) than that observed in SCMC and RD. The sensitivity of the transfected empty vector control lines did not differ markedly from that of the respective parental cell lines for any of the 4 agents.

In order to add confirmation to these observations, clonogenic survival assays were also carried out. These were less reproducible due to generally low plating efficiency but confirmed the results of the growth inhibition assay. Representative results for vincristine are shown (Figure 2E, F). The *mdm2* transfected RD cell line is significantly resistant to vincristine (67-fold) when compared with the parent line whereas there is no change in RMS.

As we observed an increased resistance to the known MDR substrate doxorubicin in 3 out of 4 *mdm2*-transfected lines, we evaluated whether the increased resistance was attributable to increased *mdr-1* or *mrp1* gene expression. Real-time PCR was used to measure expression of *mdr-1* and *mrp1* genes in the cell line panel (Figure 3A, B). Expression of *mdr-1* was undetectable in the cell lines Rh30 and RMS and their transfected variants. The line SCMC has moderate expression of *mdr-1* (as noted previously) (Cocker et al, 2000), which increases significantly following transfection with F407. Transfection of the line RD with F407 but not F373 results in a highly significant increase in *mdr-1* expression. In all the cell lines, *mrp1* expression is similar and remains unchanged following transfection. These results were confirmed using RNAse protection assay (Figure 3C). Both methods demonstrated increased expression of the *mdr-1* gene in

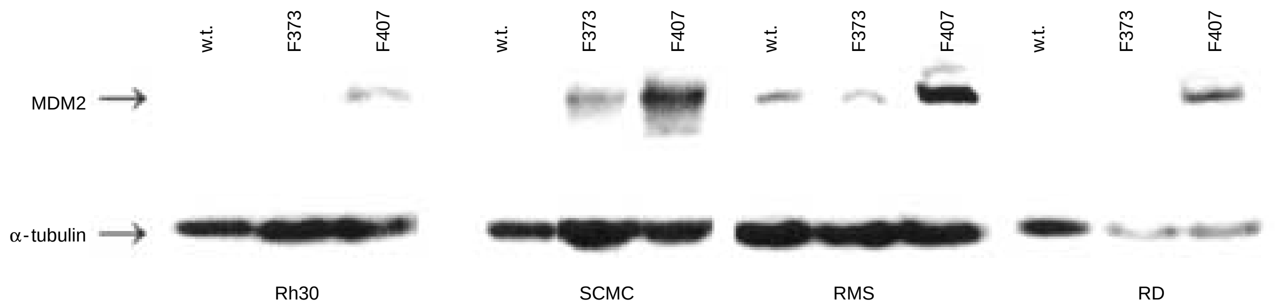

**Figure 1** Typical immunoblot for MDM2 and α-tubulin in a panel of rhabdomyosarcoma cell lines and their empty vector (F373) and *mdm2* gene (F407) transfected variants. Data are shown for one representative clone for each of the F373 empty vector and F407 *mdm2* gene transfects

 © 2001 Cancer Research Campaign

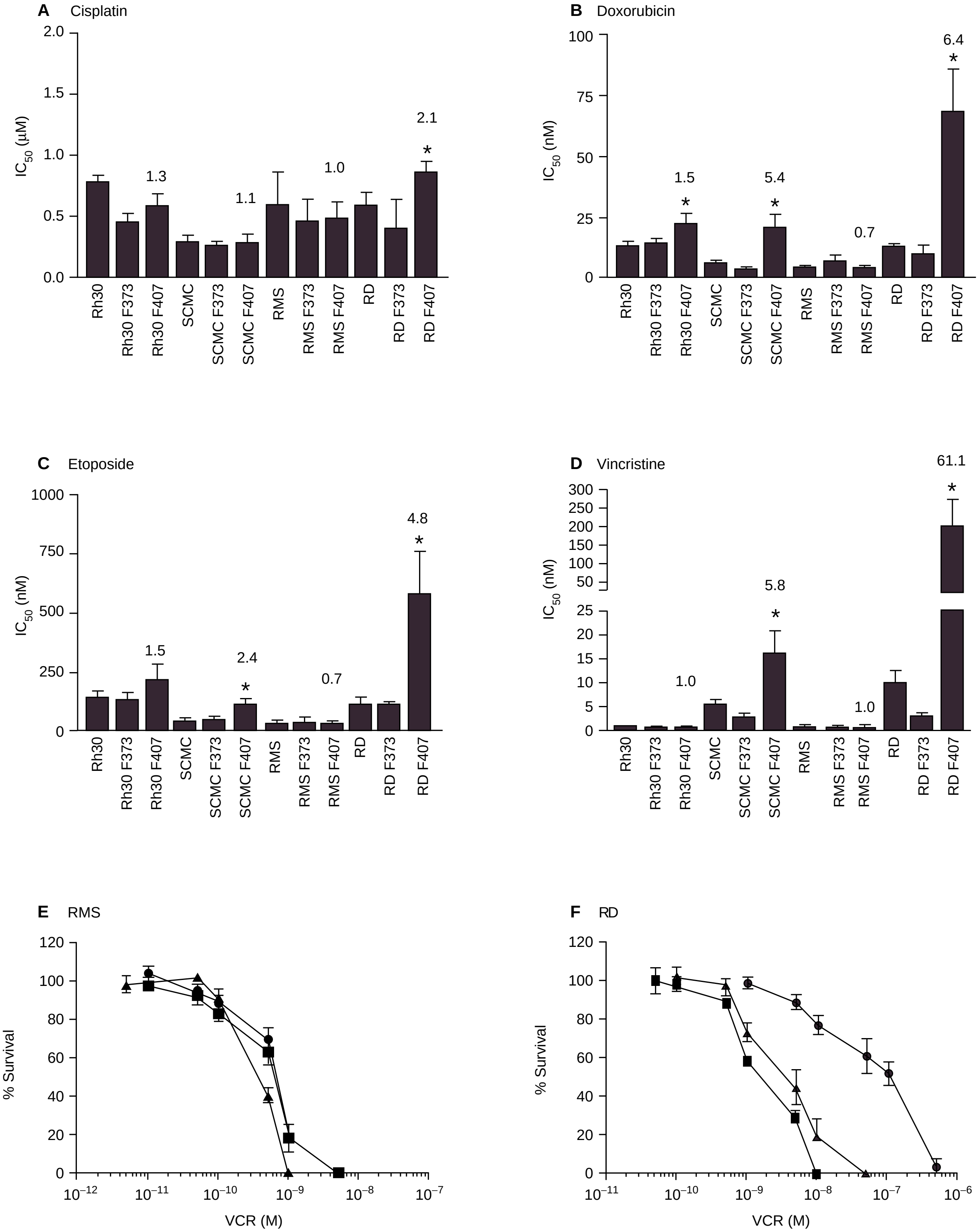

**Figure 2** Sensitivity of a panel of parent human rhabdomyosarcoma cell lines or transfected with empty vector (F373) or the *mdm2* gene (F407) to: (**A**) cisplatin, (**B**) doxorubicin, (**C**) etoposide and (**D**) vincristine. $IC_{50}$ values given are the mean + standard deviation for one representative clone as shown in Figure 1, from 3 independent experiments each containing quadruplicate measurements. Values represent fold-change in sensitivity. *indicates significantly greater than F373 transfect ($P < 0.05$) in a 2-tailed Student's *t*-test. The clonogenic assay was used to measure survival of cell lines (**E**) RMS and (**F**) RD following treatment with vincristine. Data points are the mean ± standard deviation from 3 independent experiments. ■ w.t., ▲ empty vector (F373) transfect, ● *mdm2* gene (F407) transfect

© 2001 Cancer Research Campaign

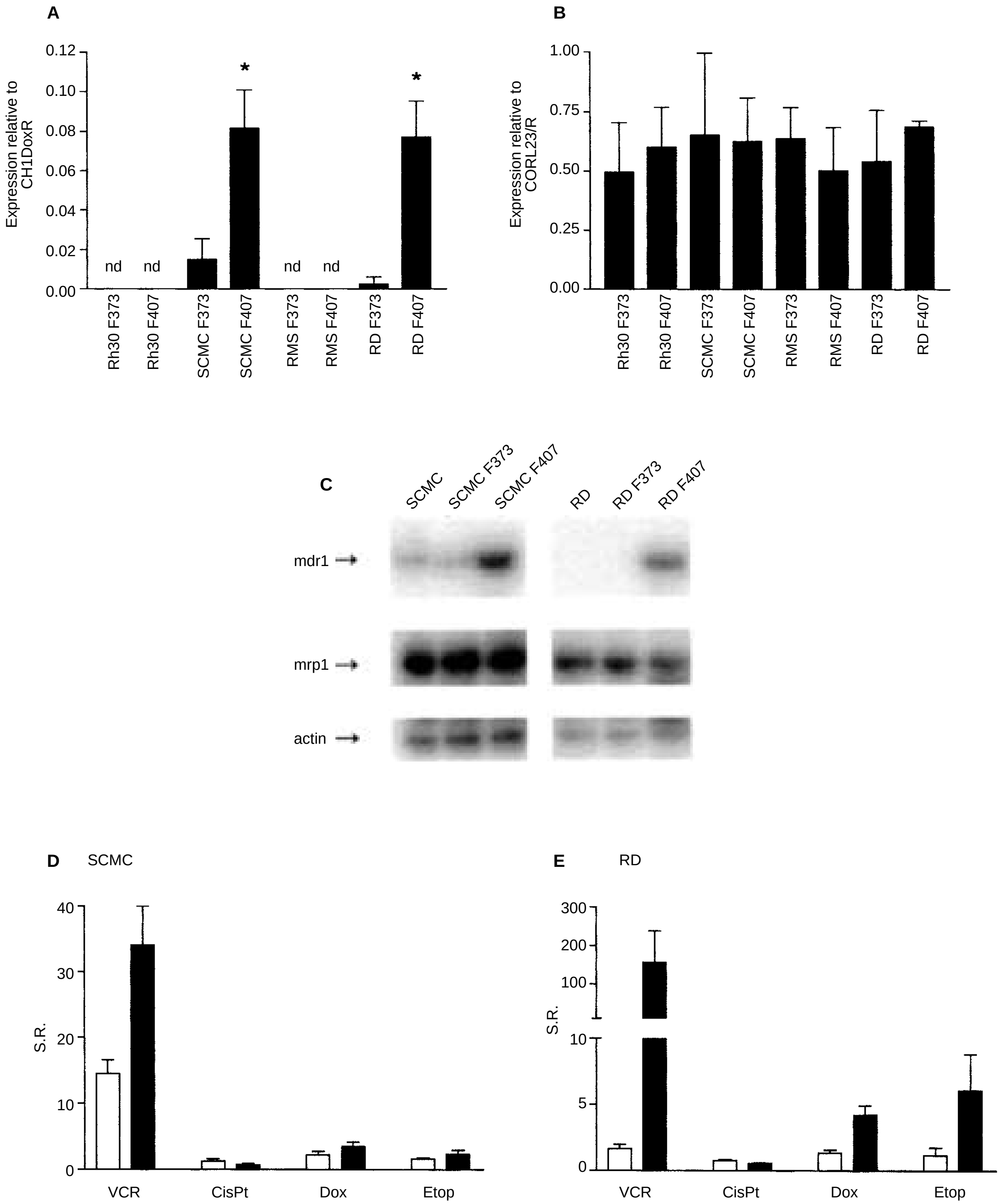

**Figure 3** Expression of MDR-related genes *mdr-1* (**A**) and *mrp1* (**B**) in human rhabdomyosarcoma cell lines transfected with empty vector (F373) or the *mdm2* gene (F407). Values shown are the mean + standard deviation from 3 independent experiments. nd = not detectable. *indicates significantly greater than F373 transfect ($P < 0.001$ in a 2-tailed Student's *t*-test). (**C**) Expression of MDR-related genes by RNAse protection assay. Modulation of sensitivity using PSC833 in (**D**) SCMC and (**E**) RD wild-type cell lines (open bars) and *mdm2* gene (F407) transfected variants (closed bars). PSC833 was added to the MTT assay immediately before addition of the cytotoxic agent at a concentration which kills less than 10% of cells (2 μM). Values given are the mean + standard deviation of 3 independent experiments each containing quadruplicate measurements. Sensitisation ratio (SR) = $IC_{50}$ in the absence of modulator/ $IC_{50}$ in the presence of modulator.

the *mdm2*-transfected variants of SCMC and RD, thus correlating with the sensitivity data. As reported previously (Cocker et al, 2000) PgP levels were too low to be detectable using immunoblotting and the C-219 antibody. Although levels were also low in the F407 transfects, detectable PgP expression was apparent in the *mdm2*-transfected variants of SCMC and RD (data not shown).

The P-gp modulator PSC833 (Naito and Tsuruo, 1997) was used to confirm that P-gp contributes to resistance in the F407- trans-

 *© 2001 Cancer Research Campaign*

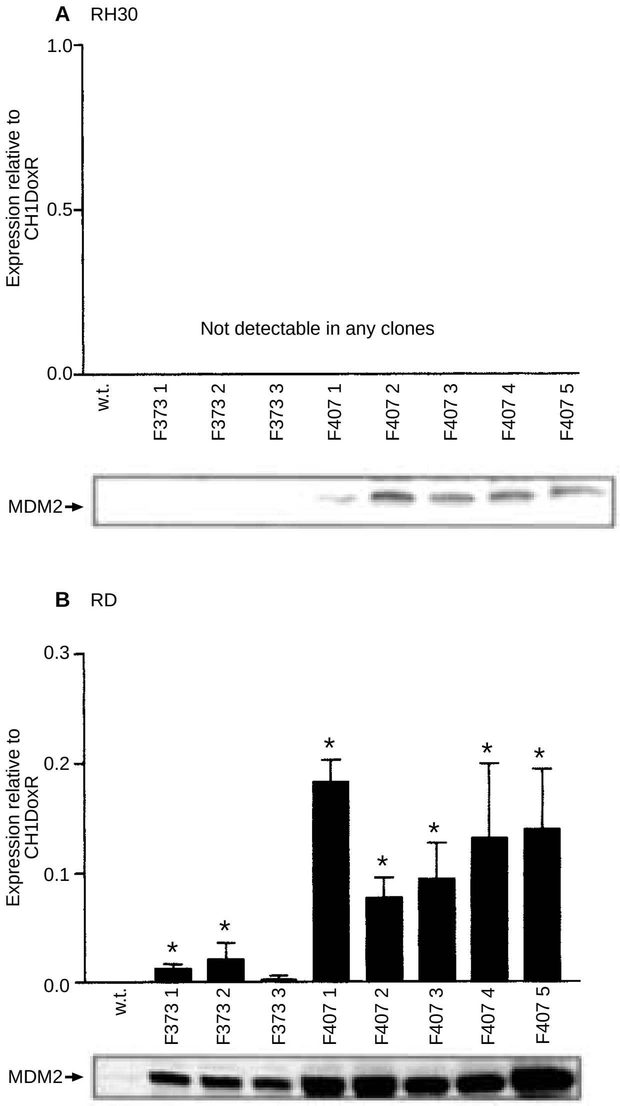

**Figure 4** Expression of the *mdr-1* gene by real-time PCR and MDM2 by immunoblotting in clones of (**A**) Rh30 and (**B**) RD. w.t. = wild type, F373 = empty vector transfected, F407 = *mdm2* gene transfected. Values are the mean + standard deviation of at least 5 independent measurements. *indicates significantly greater than wild type ($P$ < 0.001 in a 2-tailed Student's *t*-test)

fected SCMC and RD cell lines (Figure 3D, E). A sensitisation ratio greater than 1 indicates that modulation of sensitivity is taking place. PSC833 has no effect on sensitivity to cisplatin in either of the cell lines. In contrast, modulation of sensitivity to vincristine, doxorubicin and etoposide is greater in the F407-transfected lines than in their untransfected counterparts. This is particularly marked for vincristine where modulation is increased from 14-fold to 31-fold in SCMC F407 and from 1.6-fold to 156-fold in RD F407.

These results suggest that overexpression of the MDM2 protein may, in some cell lines, lead to an increase in expression of the *mdr-1* gene and P-gp, thereby conferring resistance to multiple agents. There is a possibility however, that these observations may be specific to our chosen clones. To test this we screened several transfected clones of Rh30 and RD for MDM2 expression and *mdr-1* expression (Figure 4). *mrp1* expression was also measured but was not changed in any of the clones (data not shown). In the cell line Rh30, transfection with F373 or F407 did not result in detectable expression of the *mdr-1* gene even though clear increases in MDM2 expression were observed (Figure 4A). In contrast, transfection of RD with F373 led to a small increase in expression of the MDM2 protein, concomitant with a small increase in the *mdr-1* gene, in 2 out of 3 clones. This was to a much lesser extent than following transfection with the F407 construct when both MDM2 and *mdr-1* expression were significantly increased in all 5 clones (Figure 4B).

### DISCUSSION

One previous study has suggested a possible role for MDM2 in the regulation of *mdr1* expression (Kondo et al, 1996). However, it is unclear whether this single observation is applicable to tumour types such as rhabdomyosarcoma where elevated MDM2 is occasionally observed or is dependent on aspects of the cell phenotype such as expression of *mdr1*, P53 and MDM2 itself. In order to further investigate this, we transfected the *mdm2* gene into a panel of 4 human rhabdomyosarcoma cell lines and tested their sensitivity to drugs commonly used to treat this disease, including MDR substrates.

Our results indicate that stable overexpression of MDM2 protein was achieved in all 4 cell lines and this conferred a range in response to the drugs cisplatin, doxorubicin, etoposide and vincristine. In 2 cell lines, SCMC and RD, marked resistance was observed to the P-glycoprotein substrates doxorubicin, etoposide and especially vincristine. In contrast, no effect on drug sensitivity was observed in the RMS cell line (that already overexpresses MDM2 protein) and only low-level resistance (1.5-fold) to one drug (doxorubicin) was observed in Rh30 cells. The profile of resistance in SCMC and RD suggests an MDR mechanism as the largest changes are seen with the P-gp substrates but not with cisplatin. In a few cases, transfection with the empty F373 vector also caused a small increase in expression of MDM2. This increase was much smaller than with the F407 *mdm2* construct and could reflect either clonal selection or a stress response to transfection or puromycin selection.

To aid confirmation of the mechanism of resistance, expression of the MDR genes was analysed using both real-time PCR and RNAse protection assays. Both methods demonstrated increased expression of the *mdr-1* gene in the *mdm2*-transfected variants of SCMC and RD, thus correlating with the drug sensitivity data. No significant changes were seen with the empty vector controls or in *mrp1* expression. The highly sensitive real-time PCR assay shows that the cell lines SCMC and RD both show low constitutive expression of *mdr-1* whereas Rh30 and RMS do not. This suggests that the activation of *mdr-1* expression by MDM2 may only occur when basal expression of *mdr-1* is already present. We then used the P-gp modulator PSC833 to confirm that the increased expression of *mdr-1* leads to an increase in functional P-gp. In the *mdm2*-transfected variants of SCMC and RD, resistance to vincristine, doxorubicin and etoposide could be modulated using PSC833 whereas sensitivity to cisplatin remained unchanged.

To confirm that our observations were not clone-dependent we screened a series of transfected clones for MDM2, *mdr-1* and *mrp1* expression. No changes in *mrp1* expression were seen, indicating that MDM2 has no influence on this gene. In the cell line Rh30 transfection with F373 or F407 did not result in detectable expression of the *mdr1* gene even though clear increases in MDM2

*© 2001 Cancer Research Campaign*

protein were detected with the F407 transfects. In contrast, transfection of the F407 construct into RD cells resulted in overexpression of MDM2 protein and a significant increase in the *mdr1* gene in all 5 clones. These observations support the hypothesis that increases in *mdr-1* expression are only seen when basal expression is present, as in RD and SCMC. Reports suggest that MDM2 may function as a transcription factor (Leveillard and Wasylyk, 1997). If MDM2 is able to influence transcription of the *mdr-1* gene this may only occur when the promoter region is already active.

Of interest was that, of the 2 cell lines where high levels of *mdm2* caused an increase in drug resistance, SCMC possesses wild-type p53 while RD is mutant. We also evaluated the selected clones for constitutive p53 expression using an antibody (DO-1) which detects both wild-type and mutant forms. The 3 p53 mutant lines (RD, RMS and Rh30) all showed high constitutive levels of p53 in empty vector F373 control and F407 *mdm2* transfects (data not shown). In contrast, the F407 *mdm2* transfects of SCMC showed higher constitutive p53 expression than either the vector controls or parent line. It would be of interest to evaluate relationships between *mdm2* and induction of *mdr-1* in a p53 null rhabdomyosarcoma cell line such as Rh4 (Taylor et al, 2000). The changes in drug sensitivity observed in the mutant p53 RD cell line may relate to the recent observation of mutant p53 stabilising MDM2 (Peng et al, 2001).

In conclusion, we have confirmed that overexpression of MDM2 may increase the expression of the *mdr-1* gene. This may be dependent on the presence of *mdr-1* transcription before transfection takes place. MDM2 overexpression often occurs during the development of malignancy, especially in soft tissue sarcomas. These data are in agreement with one previous report where *mdm2* was transfected into a human glioblastoma cell line and selected using G418 (Kondo et al, 1996). However, in contrast to our studies, no sensitivity data are reported for empty vector control clones. A recent study found amplification of the *mdm2* gene in 2/20 cases of rhabdomyosarcoma (Taylor et al, 2000). In our own studies of *mdm2* gene expression in 28 rhabdomyosarcoma clinical samples, 4 (14%) expressed high levels as measured by real-time PCR (unpublished observations). It is therefore conceivable that in some tumours, *mdr-1* expression may be increased before any treatment takes place and this may influence the management of disease. These data also suggest that inhibition of MDM2 function (eg. by small molecules or antisense oligonucleotides) may be used in some rhabdomyosarcoma tumours to prevent resistance to MDR substrates. Our models provide further evidence for a relationship between MDM2 and *mdr-1*. However, in our initial study of 28 clinical samples we did not observe high *mdr-1* expression in the 4 samples with high *mdm2* expression (data not shown). A larger study in clinical samples both at diagnosis and after chemotherapy or at relapse is required to confirm the clinical significance.

### ACKNOWLEDGEMENTS

This work is supported by an Institute of Cancer Research studentship. We thank J Heward for technical guidance and Novartis Pharmaceuticals for donating PSC833.

### REFERENCES

Ashcroft M and Vousden KH (1999) Regulation of p53 stability. *Oncogene* **18**: 7637–7643

Barrand MA, Heppell-Parton AC, Wright KA, Rabbitts PH and Twentyman P (1994) A 190-kDa protein overexpressed in non-P-glycoprotein containing multidrug resistant cells and its relationship to the MRP gene. *J Natl Cancer Inst* **86**: 110–117

Bordow SB, Habrer M, Madafiglio J, Cheung B, Marshall GM and Norris MD (1994) Expression of the multidrug resistance-associated protein (MRP) gene correlates with amplification and overexpression of the N-myc oncogene in childhood neuroblastoma. *Cancer Research* **54**: 5036–5040

Chan HSL, Thorner PS, Haddad G and Ling V (1990) Immunohistochemical detection of P-glycoprotein – Prognostic correlation in soft tissue sarcoma of childhood. *J Clin Oncol* **8**: 689–704

Chin K-V, Ueda K, Pastan I and Gottesman MM (1992) Modulation of activity of the promoter of the human MDR1 gene by Ras and p53. *Science* **255**: 459–462

Cocker HA, Pinkerton CR and Kelland LR (2000) Characterization and modulation of drug resistance of human paediatric rhabdomyosarcoma cell lines. *British Journal of Cancer* **83**: 338–345

Enzinger FM and Weiss SW (1995) *Soft tissue tumors*. The CV Mosby Co: St Louis

Hobbs S, Jitapakdee S and Wallace JC (1998) Development of a bicistronic vector driven by the human polypeptide chain elongation factor 1α-promotor for creation of stable mammalian cell lines that express very high levels of recombinant proteins. *Biochemical and Biophysical Research Communications* **252**: 368–372

Keleti J, Quezado MM, Abaza MM, Raffeld M and Tsokos M (1996) The MDM2 oncoprotein is overexpressed in rhabdomyosarcoma cell lines and stabilizes wild-type p53 protein. *American Journal Pathology* **149**: 143–151

Kondo S, Kondo Y, Hara H, Kaakaji R, Peterson JW, Morimura T, Takeuchi J and Barnett GH (1996) mdm2 gene mediates the expression of mdr1 gene and P-glycoprotein in a human glioblastoma cell line. *British Journal of Cancer* **74**: 1263–1268

Kuttesch JF, Parham DM, Luo X, Meyer WH, Bowman L, Shapiro DN, Pappo AS, Crist WM, Beck WT and Houghton PJ (1996) P-Glycoprotein expression at diagnosis may not be a primary mechanism of therapeutic failure in childhood rhabdomyosarcoma. *Journal Clinical Oncology* **14**: 886–900

Leveillard T and Wasylyk B (1997) The MDM2 C-terminal region binds to TAF(II)250 and is required for MDM2 regulation of the cyclin A promoter. *J Biological Chemistry* **272**: 30651–30661

Li ZH, Zhu YJ and Lit XT (1997) Wild-type p53 gene increases MDR1 gene expression but decreases drug resistance in an MDR cell line KBV200. *Cancer Letters* **119**: 177–184

Meddeb M, Valent A, Danglot G, Nguyen VC, Duverger A, Fouquet F, Terrier-Lacombe MJ, Oberlin O and Bernheim A (1996) MDM2 amplification in a primary alveolar rhabdomyosarcoma displaying a t(2;13)(q35;q14). *Cytogenetics Cell Genetics* **73**: 325–330

Mosmann T (1983) Rapid colorimetric assay for cellular growth and survival: application to proliferation and cytotoxicity assays. *Journal Immunological Methods* **65**: 55–63

Naito M and Tsuruo T (1997) New multidrug resistance reversing drugs, MS-209 and SDZ PSC833. *Cancer Chemother Pharmacol* **40**: S20–S24

Peng Y, Chen L, Li C, Lu W, Agrawal S and Chen J (2001) Stabilization of the MDM2 oncoprotein by mutant p53. *Journal Biological Chemistry* **276**: 6874–6878

Sharp SY, Rowlands MG, Jarman M and Kelland LR (1994) Effects of a new antioestrogen, idoxifene, on cisplatin- and doxorubicin-sensitive and resistant human ovarian carcinoma cell lines. *British Journal of Cancer* **70**: 409–414

Suzuki A, Toi M, Yamamoto Y, Saji S, Muta M and Tominaga T (1998) Role of MDM2 overexpression in doxorubicin resistance of breast carcinoma. *Japan Journal Cancer Research* **89**: 221–227

Tanner B, Hengstler JG, Laubscher S, Meinert R, Oesch F, Weikel W, Knapstein PG and Becker R (1997) Mdm2 mRNA expression is associated with survival in ovarian cancer. *Int J Cancer* **74**: 438–442

Taylor AC, Shu L, Danks MK, Poquette CA, Shetty S, Thayer MJ, Houghton PJ and Harris LC (2000) p53 mutation and MDM2 amplification frequency in pediatric rhabdomyosarcoma tumors and cell lines. *Medicine Pediatric Oncology* **35**: 96–103

Thottassery JV, Zambetti GP, Arimori K, Schuetz EG and Schuetz JD (1997) p53-dependent regulation of MDR1 gene expression causes selective resistance to chemotherapeutic agents. *Proc Natl Acad Sci* **94**: 11037–11042

Zhou M, Gu L, Jurickova I, Yarrington AK, Absbire T, Homans A, Yeager AM and Findley HW (1997) Elevated MDM2 protein expression in pediatric acute lymphoblastic leukemia (ALL) is associated with in vitro resistance to adriamycin and decreased duration of first remission (CR1). *Blood* **90**: 824–824

*© 2001 Cancer Research Campaign*
